# Design and force analysis of end-effector for plug seedling transplanter

**DOI:** 10.1371/journal.pone.0180229

**Published:** 2017-07-05

**Authors:** Zhuohua Jiang, Yang Hu, Huanyu Jiang, Junhua Tong

**Affiliations:** 1 College of Biosystems Engineering and Food Science, Zhejiang University, Hangzhou, China; 2 Key Laboratory of Equipment and Informatization in Environment Controlled Agriculture, Ministry of Agriculture, Hangzhou, China; 3 Faculty of Mechanical Engineering & Automation, Zhejiang Sci-Tech University, Hangzhou, China; Universita degli Studi della Tuscia, ITALY

## Abstract

Automatic transplanters have been very important in greenhouses since the popularization of seedling nurseries. End-effector development is a key technology for transplanting plug seedlings. Most existing end-effectors have problems with holding root plugs or releasing plugs. An efficient end-effector driven by a linear pneumatic cylinder was designed in this study, which could hold root plugs firmly and release plugs easily. This end-effector with four needles could clamp the plug simultaneously while the needles penetrate into the substrate. The depth and verticality of the needles could be adjusted conveniently for different seedling trays. The effectiveness of this end-effector was tested by a combinational trial examining three seedling nursery factors (the moisture content of the substrate, substrate bulk density and the volume proportion of substrate ingredients). Results showed that the total transplanting success rate for the end-effector was 100%, and the root plug harm rate was below 17%. A force measure system with tension and pressure transducers was installed on the designed end-effector. The adhesive force *F*_*L*_ between the root plug and the cell of seedling trays and the extrusion force *F*_*K*_ on the root plug were measured and analyzed. The results showed that all three variable factors and their interactions had significant effects on the extrusion force. Each factor had a significant effect on adhesive force. Additionally, it was found that the end-effector did not perform very well when the value of *F*_*K*_/*F*_*L*_ was beyond the range of 5.99~8.67. This could provide a scientific basis for end-effector application in transplanting.

## Introduction

With the popularization of seedling nurseries, transplantation has become a very important activity in greenhouses. Manual transplantation in greenhouses is labor-intensive and costly, and growers have become increasingly interested in using automatic seedling transplanters. Since the first robotic transplanter was designed in 1987[1], much progress has been achieved [2–6]. Research on automatic transplanters has mainly focused on mechanical engineering[7, 8], machine vision[9, 10] and control engineering[11, 12]. Design of an end-effector is key for transplanting plug seedlings [13–15]. Classified by the picking action of fingers, end-effectors are divided into two complementary forms, the clamp form[7, 15] and slide form[8, 16]. Usually fingers of clamp end-effectors are naked and fingers of slide end-effectors have sleeves. There main difference between these two forms is the picking action. For grasping a plug seedling, these two forms have differernt actions. Clamp end-effectors need to approach the seedling from the top, and keep going down until the fingers stab into the substrate and reach a certain depth, then the fingers clamp to the center of root plugs. The verticality of clamp end-effector fingers would be changed after clampping. Slide end-effectors with fingers retracting into corresponding sleeves also need to approach the seedling from the top, but stop going down when the sleeves reach the surface of the root plugs, then the fingers extend out of sleeves and stab into the substrate. The verticality of slide end-effector fingers would not be changed during the transplanting process. Compared with slide end-effectors, clamp end-effectors have better performance in grasping and holding plugs but have problems in releasing plugs. Sometimes, root plugs strongly adhere to the fingers and cannot be released successfully by gravity. On the contrary, slide end-effectors release plugs more effectively by the relative motion of needles and their sleeves but do not perform very well in grasping and holding plugs. To combine these advantages, an end-effector driven by a linear pneumatic cylinder was designed. This end-effector could clamp the plug with four needles simultaneously when the needles are stabbing into the nutritional substrate.

Plug seedlings are the working object of end-effectors, and the cohesion to the root plug has a significant impact on transplantation results. One of the most important morphological quality criteria for plug seedlings is to have a firm and cohesive root plug, which is good for transplantation. The density and uniformity of the root system mainly ensure high cohesion to the root plug, as well as the moisture content, the substrate bulk density and the volume proportion of substrate ingredients (peat: vermiculite: perlite)[17]. In recent years, researchers have realized that innovations of end-effectors were not enough to promote automatic transplantation and began to combine the research with root plugs [9, 18–20]. Usually, the extrusion force that end-effector put on root plug and the adhesive force between the root plug and plug cell’s inwall were used to estimate the root plug. The extrusion force has a great responsibility to the transplantation performance. Root plug would be cracked if the extrusion force was too large. In contrast, plug seedling couldn’t be lifted out of the plug cell if the extrusion force was too small to overcome the adhesive force. The adhesive force was related to the conditions of growth media and material characteristics of plug tray. However, published studies separated the studies of the root plug from the transplanting process. Plug seedlings for the extrusion test were carefully moved out from plug trays manually. For the adhesive test, plug seedlings were usually grasped at the stems and pulled out from the plug cell. The relationship between these two forces and the transplantation performance of the same plug seedling could not be analyzed well. There were no existing systems to measure these two forces directly or indirectly during the end-effector’s transplantation movements. To solve this problem, a force measurement system was installed on the designed end-effector with a tension transducer and pressure transducer. During the practical transplanting process, the two forces could be recorded and calculated directly through the transducers.

The present study aims to design an efficient end-effector for automatic transplanters in greenhouses and analyze the extrusion and adhesive forces during the transplanting process. Root system is not a agronomic factor, and the other three are agronomic factors that could be controled during nursery. The density and uniformity of the root system are also influenced by the three agronomic factors. In this manuscript, we just want to anslyze the agronomic factors. The specific objectives of this study are: (1) to design the structure of end-effector, (2) to establish a force measurement system to record extrusion and adhesive forces based on the designed end-effector, (3) to verify the adaptability and efficiency of the designed end-effector through a combinational experiment on the moisture content of substrate, the substrate bulk density and the volume proportion of substrate ingredients, and (4) to analyze the extrusion and adhesive forces during the transplanting process.

## Materials and methods

### Structure design of the end-effector

**Fig 1:**
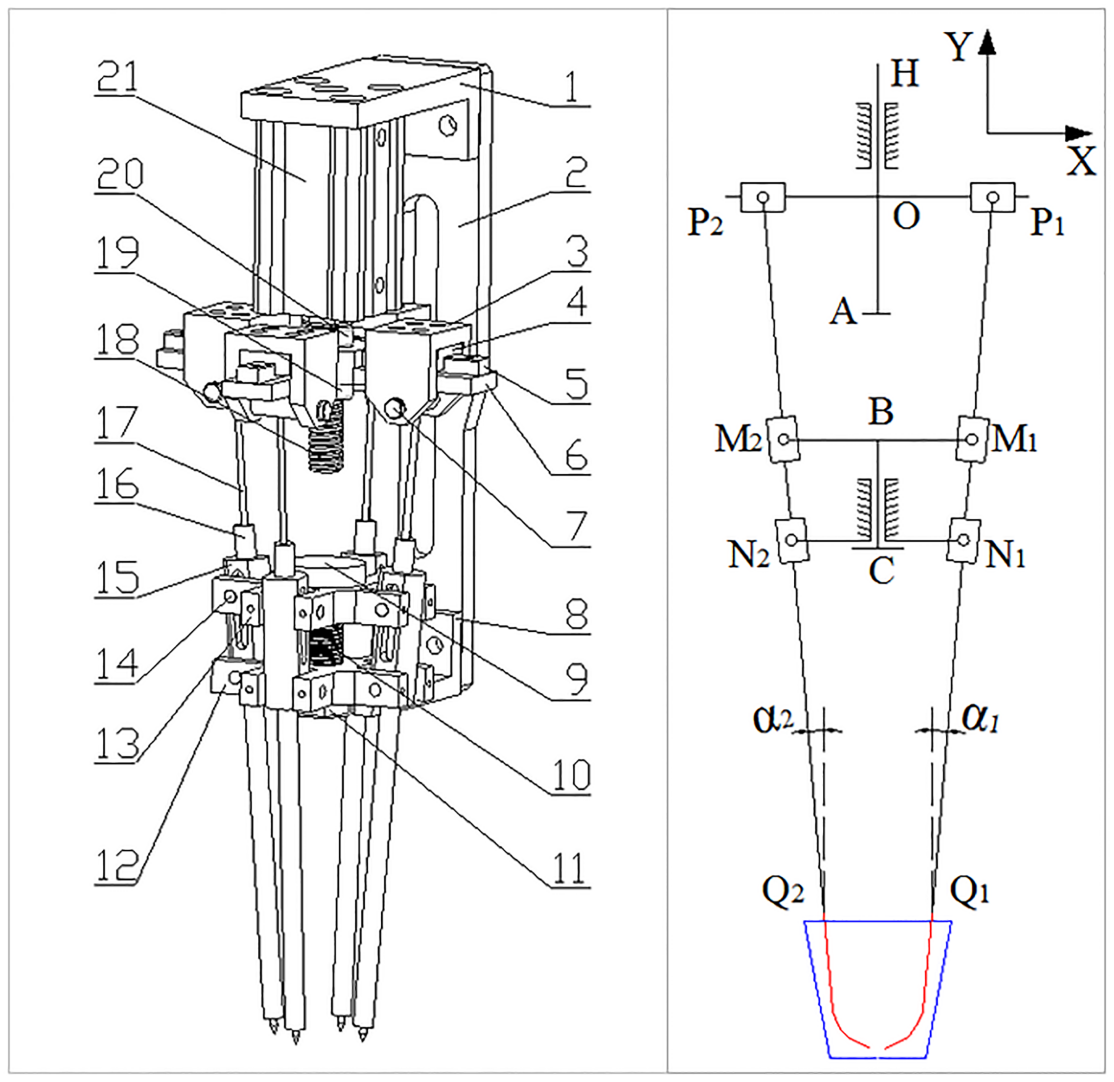
The structure of the end-effector. 1. Upper Bracket, 2. Mounting Bracket, 3. Slide Sleeve, 4. Slider, 5. Sliding Guide, 6. Upper Cross Plane, 7. Spindle, 8. Lower Bracket, 9. Bolt, 10. Spring, 11. Hexagon Nut 1, 12. Bottom Cross Plane, 13. Middle Cross Plane, 14. Short Pin, 15. Rotation Part, 16. Slide sleeve, 17. Needle, 18. Thread Rod, 19. Hexagon Nut 2, 20. Hexagon Nut 3, 21. Pneumatic Cylinder.

A good end-effector of transplantation, besides being reliable, should be able to effectively penetrate, grasp, hold, and release a wide variety of growth media with minimum damage to the roots. Other guidelines were that the end-effector should be structured simply and powered by low-cost actuators; accommodate a wide variety of plugs in different sizes of containers; and avoid plug aerial portion and operate on the root zone portion [13]. However, most existing end-effectors were clamp forms or slide forms. Clamp end-effectors have problems in releasing plugs, and slide end-effectors have problems in grasping and holding plugs. In contrast, slide end-effectors performed well in releasing plugs and clamp end-effectors performed well in grasping and holding plugs. Based on these guidelines, an effective end-effector combined the advantages of clamp forms and slide forms was shown in Fig 1A. There are four symmetrically slanted needles, and each needle slides within a sleeve. The needles are actuated by a double-acting pneumatic cylinder. The depth into growth media and the verticality of needles could be adjusted easily for different plug trays and cell sizes. The depth could be adjusted by changing the position of the Upper Cross Plane, which is fixed to a Thread Rod by Hexagon Nuts 2 and 3. The verticality of needles could be adjusted through the Rotation Part. Screwing Hexagon Nut 1 changes the position of the Middle Cross Plane, thus the Rotation Part could rotate and change the verticality. Fig 1B is the planar kinematic sketch of the designed end-effector. The red curves are the trajectories of the two needle cusps in the diagonal direction.

**Fig 2:**
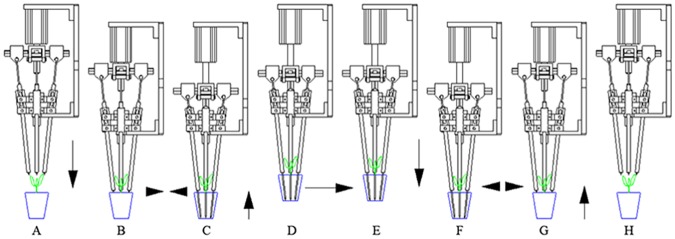
Process for transplanting one plug seedling.

Fig 2 shows the process for transplanting one plug seedling. At the beginning of a transplanting cycle, the end-effector, with its needles retracted (Fig 2A), would approach a plug from the top (Fig 2B) and extend the needles to penetrate into the growth media (Fig 2C). After the Thread Rod knocked against the Bolt, the needles kept penetrating and started clamping the root zone portion of the plug. Then, the plug would be lifted out of the cell (Fig 2D) and transported to a growing tray (Fig 2E). After getting the position right above the empty cell that needed to be filled, the end-effector would lower the plug into an empty cell in the growing tray (Fig 2F) and retract the needles to release the plug (Fig 2G). The transplanting cycle is completed by leaving the growing tray (Fig 2H). With the relative motion between needles and corresponding sleeves, the sleeves would push the plug into the cell in an upright position. This end-effector combined the advantages of clamp form and slide form without increasing the time consumption.

### Force measurement system

**Fig 3:**
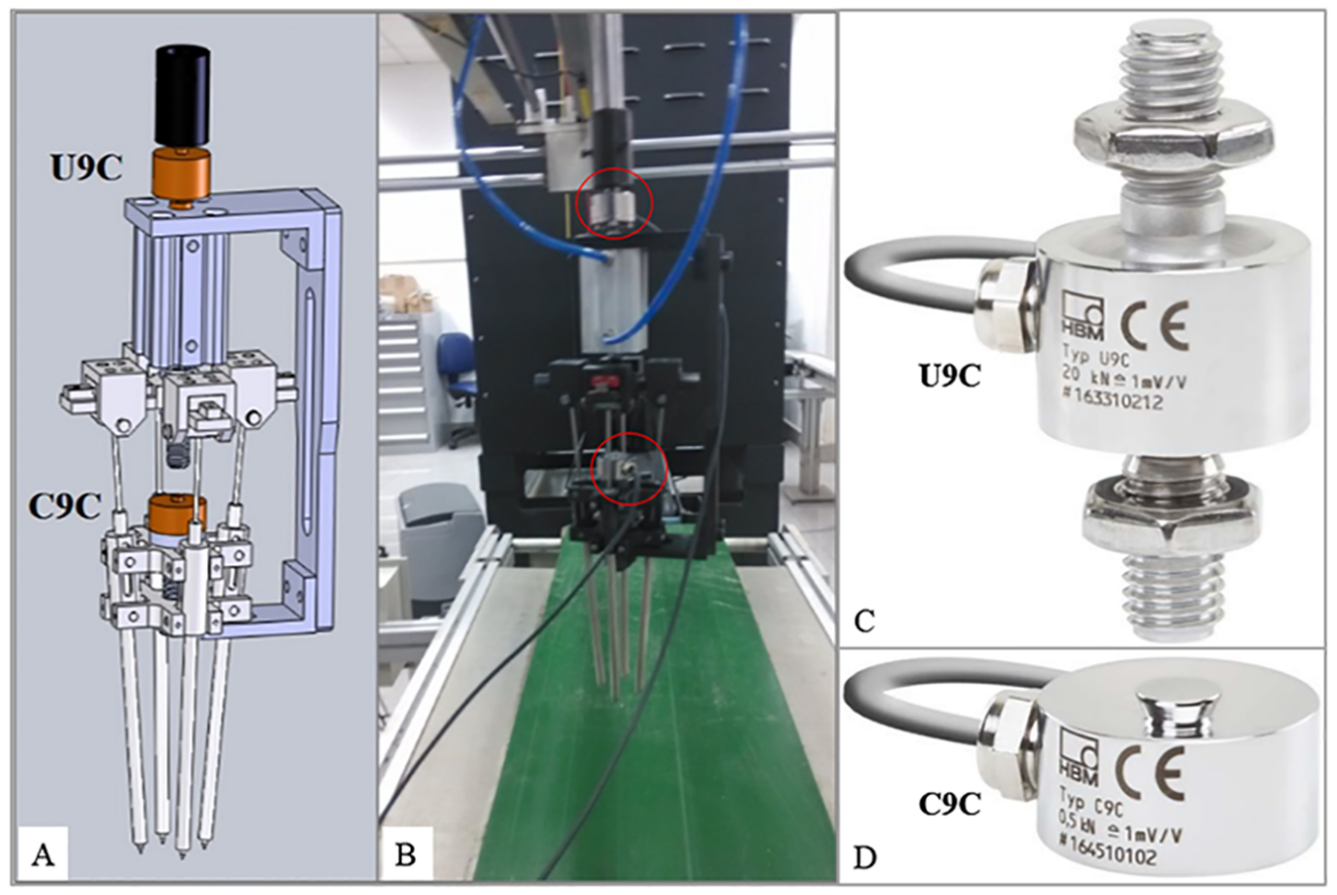
Force measurement system.

**Fig 4:**
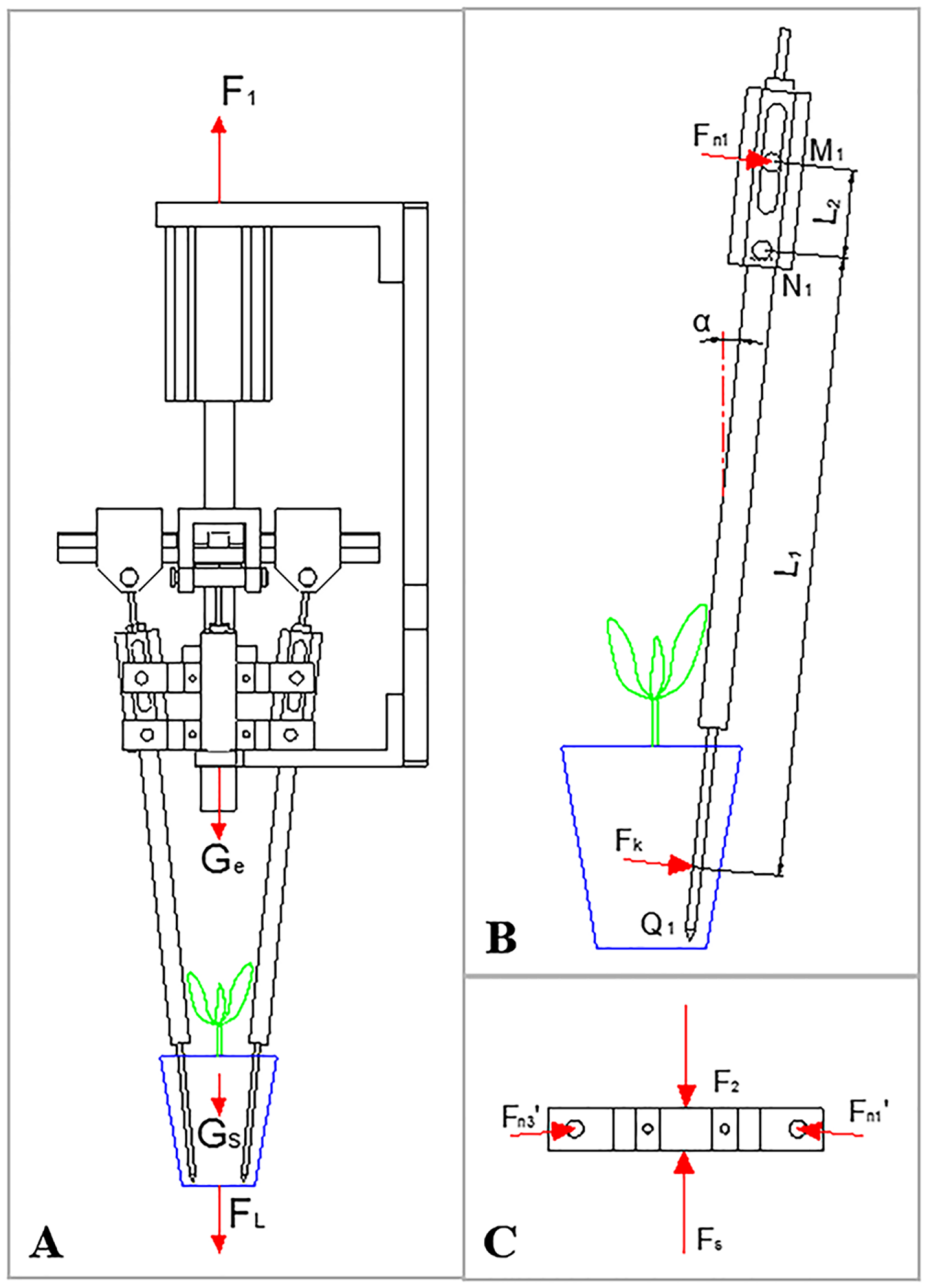
Force analysis of the measurement system.

Based on the designed end-effector, a force measurement system was established to record the extrusion and adhesive forces. As shown in Fig 3, a tension transducer (HBM, U9C/100N) was installed between the end-effector and mechanical arm, and a pressure transducer (HBM, C9C/100N) was fixed on the bolt of end-effector. The output signals were amplified by two transmitters (LONGTEC and TR200H). The amplification factor was about 100. Then, the amplified signals were collected and analyzed by a portable data collector and analyzer (AVANT MI-7008), with the signal size of 8192 and the sampling frequency of 2560. All the data were stored in a computer for processing and analysis. Fig 4 displays the forces in the measurement system, and four equations can be obtained as follows:

$$F_{1}=F_{L}+\left(G_{e}+G_{s}\right)$$ (1)

$$F_{2}=F_{s}+4F_{n1}^{\prime }\text{sin}\alpha$$ (2)

$$F_{n1}=F_{n1}^{\prime }$$ (3)

$$F_{k}L_{1}=F_{n1}L_{2}$$ (4)

where *F*_*1*_ is the tension on U9C transducer when the end-effector is lifting a plug seedling, *F*_*L*_ is the adhesive force between the root plug and plug cell inwall, *G*_*e*_ is the total gravity of end-effector, *G*_*s*_ is the gravity of a plug seedling, *F*_*2*_ is the stress on the C9C transducer when the end-effector is clamping a root plug, *F*_*s*_ is the total resistance when the end-effector is clamping without a root plug, *F*_*n1*_ ($F_{n1}^{\prime }$) is the acting force between the Rotation Part and Short Pin, *F*_*k*_ is the extrusion force on a root plug, *L*_*1*_ is the distance between the acting point of *F*_*k*_ (1/3 of the stabbing depth from the needle cusp) and the rotation center (*N*_*1*_), *L*_*2*_ is the distance between the acting point of *F*_*n1*_ and the rotation center. The applied force *F*_*k*_ and *F*_*n1*_ act as torques. Then, the adhesive force *F*_*L*_ and the extrusion force *F*_*k*_ are calculated as follows:

$$F_{L}=F_{1}-\left(G_{e}+G_{s}\right)$$ (5)

$$F_{k}=\frac{L_{2}\left(F_{2}-F_{s}\right)}{4L_{1}\text{sin}\alpha }$$ (6)

### Transducer calibration

**Fig 5:**
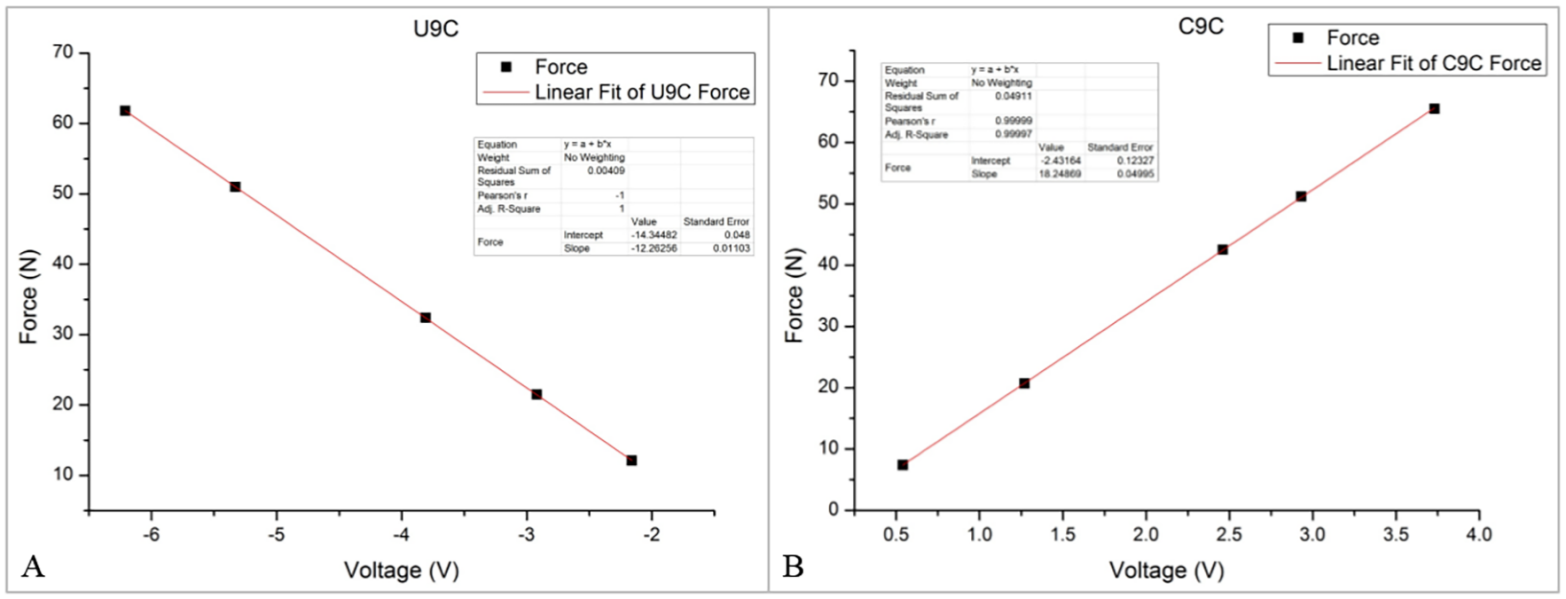
Linear fit for the U9C and C9C transducers.

U9C and C9C transducers designed for measuring static and dynamic forces were used in the measurement system. The nominal forces were the same as 100 N, and the limit forces were 200% of nominal forces (200 N). The relative reproducibility and repeatability errors without rotation were smaller than 0.2% of nominal forces (0.2 N). The nominal temperature range was -10°C to +70°C. After the transmitters and signal collecting device configured as mentioned above, the transducers were calibrated on a standard electric universal testing machine (CMT4204). And the accuracy was 0.1 N. Force values and the corresponding voltage values are shown in Table 1. Fig 5 shows the liner fit for the U9C and C9C transducers. The linear fitting equations are as follows:

$$F_{U}=-12.26V_{U}-14.34$$ (7)

$$F_{C}=18.25V_{C}-2.43$$ (8)

where *F*_*U*_ is the tension on the U9C transducer, *V*_*U*_ is the voltage measured by the U9C transducer, *F*_*C*_ is the stress on the C9C transducer, and *V*_*C*_ is the voltage measured by the C9C transducer.

**Table 1:** Force values and the corresponding voltage values.

U9C	Voltage/V	-2.16	-2.92	-3.81	-5.33	-6.21
	Force/N	12.1	21.5	32.4	51.0	61.8
C9C	Voltage/V	0.54	1.27	2.46	2.93	3.73
	Force/N	7.4	20.7	42.5	51.2	65.5

### Performance tests of the designed end-effector

**Fig 6:**
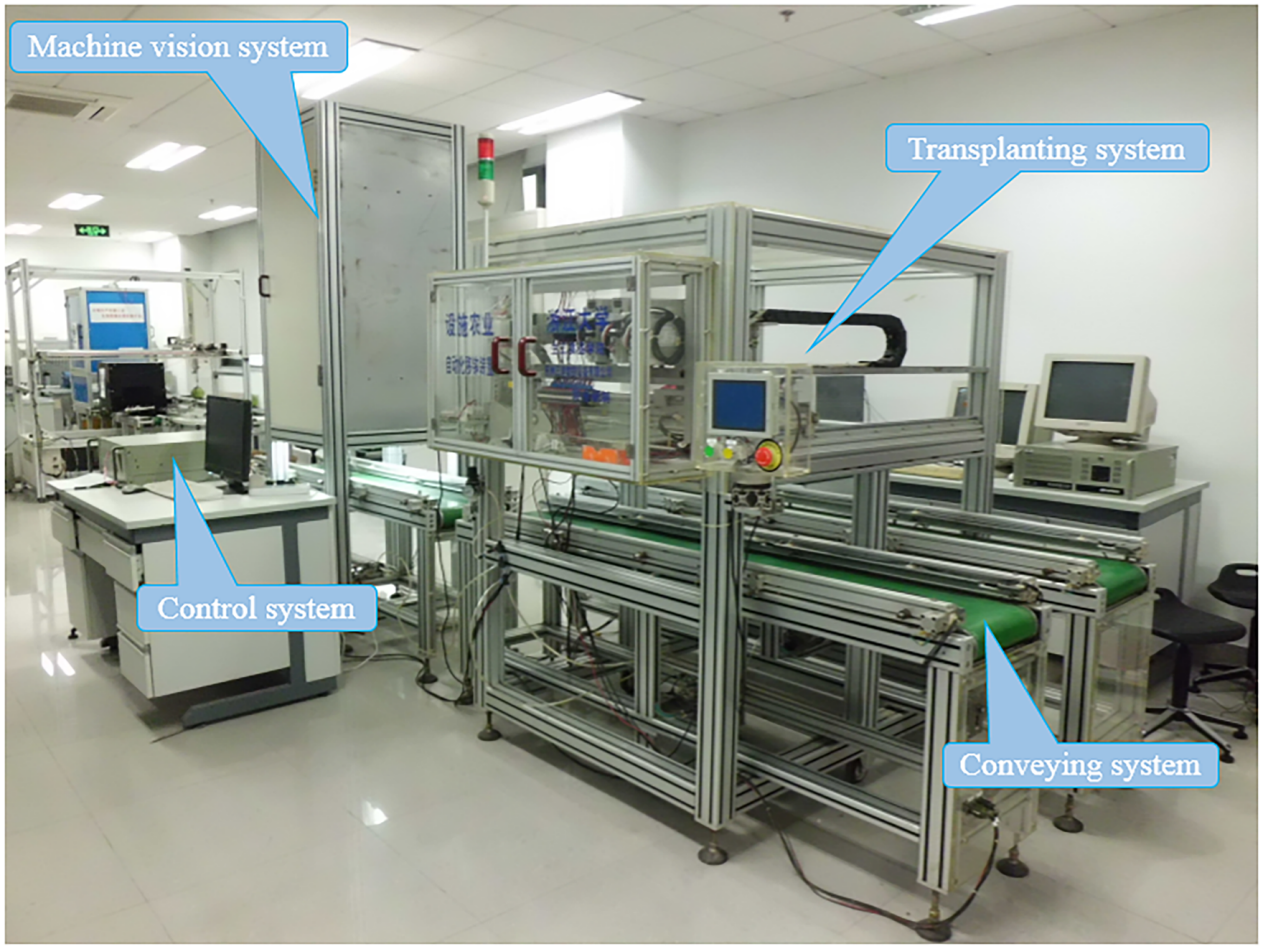
Transplanting platform with the designed end-effector.

The interaction experiment is an effective measurement to assay the comprehensive effect of multiple factors. To test the adaptability and efficiency of the designed end-effector, an interacting effect trial on the moisture content of the substrate, substrate bulk density and volume proportion of substrate ingredients was designed. Each factor had three levels. Table 2 shows the factors and levels of the interaction experiment. To meet the requirements of seedling nurseries, the substrate porosity were greater than 85% [21], and all the water potentials were over -10 kPa [17]. On March 4, 2016, cucumber seeds (JinPei 99F1, ZYANHN) were sowed in nine 6×12 trays (overall dimension 280×540*mm*) and nursed in the greenhouse at Zhejiang University. On the 14th day after sowing, the test was carried out on a transplanting platform (shown in Fig 6). The nine trays were divided into three groups and numbered individually. Trays in the same group had the same volume proportion (peat: vermiculite: perlite), but the average substrate bulk density in each cell increased from the former one in numbers. A total of 504 g, 612 g and 720 g nutritional substrate were evenly spread in the three trays of each group (7 g, 8.5 g and 10 g substrate per cell). The volume of the plug cell was 38.2 ml. The first group included Trays 1 to 3, which were filled with a peat: vermiculite: perlite mixture (6:3:1, v/v/v) [16]. The second group included Trays 4 to 6, which were filled with a peat: vermiculite: perlite mixture (6:2:2, v/v/v) [22]. The third group included Trays 7 to 9, which were filled with a peat: vermiculite: perlite mixture (7:2:1, v/v/v) [23]. All the three volume proportions are frequently used in seedling nurseries. Each tray was divided into three regions evenly along the long side of the tray. All of the trays were watered plentifully (water began to drip from the hole at the bottom) every morning till the last time. Region 1 and Region 2 were last watered four days and two days before the test beginning day respectively, and Region 3 was last watered in the morning of the test beginning day. The relative moisture content of the three regions in the same tray was different when the test was carried out (78%, 80%, and 82%). Ten plug seedlings in each region were randomly chosen for transplanting, and the results of transplantation were recorded, along with the signals of transducers. The working pressure of the pneumatic cylinder (SMC CQ2A20-40DM) was set as 0.44 MPa. The diameters of the needles were 2 mm. The stabbing depth of the needles was 40 mm, and the needles stabbed into the substrate at a 4° vertical angle. The distance between the two needle cusps in the diagonal direction was 36 mm before clamping and then contracted to 16 mm after clamping. The end-effector lifted plug seedlings at a speed of 0.1 m/s and moved at a speed of 1 m/s as set. The average time cost for transplanting one plug seedling is 2.8 s.

**Table 2:** Factors and levels of the full interaction experiment.

Levels	Factors		
	Moisture content A	Bulk density B (g/ml)	Volume proportion C (peat: vermiculite: perlite)
**1**	82%	0.183	6:3:1
**2**	80%	0.223	6:2:2
**3**	78%	0.262	7:2:1

## Results and discussion

### Transplanting performance of end-effector

Table 3 lists the treatment and seedling germination rate. All the germination rates are over 90%. Some germination rates are as high as 98.6% (Tray 1, Tray 5 and Tray 8), which means that each tray of those only have one empty cell. Table 4 shows the transplanting results of the plug seedlings, which are classified in three kinds. Success results show that the plug seedlings were successfully lifted from the high-density trays, transferred to the low-density trays and released in the empty cell. If any step of the three procedures failed, the transplanting results would be identified as a fail. Sometimes the root plugs of successfully transplanted seedlings were cracked during transplantation, and these results were identified as harm. The success rate *Rs* and harm rate *Rh* are calculated as follows:

$$Rs=\frac{\text{s}}{n}\times 100\%$$ (9)

$$Rh=\frac{h}{n}\times 100\%$$ (10)

where *s* is the number of successfully transplanted seedlings, *n* is total number of transplanted seedling, and *h* is the number of harmfully transplanted seedlings.

**Table 3:** Tray treatment and seedling germination rate.

Tray number	Volume proportion	Bulk density (g/ml)	Germination rate /%
**1**	6:3:1	0.183	98.6
**2**	6:3:1	0.223	94.4
**3**	6:3:1	0.262	94.4
**4**	6:2:2	0.183	91.7
**5**	6:2:2	0.223	98.6
**6**	6:2:2	0.262	93.1
**7**	7:2:1	0.183	93.1
**8**	7:2:1	0.223	98.6
**9**	7:2:1	0.262	95.8

**Table 4:** Transplantation results for plug seedlings.

Factors	Levels	Total number	Success number	Harm number	Success rate/%	Harm rate/%
**Moisture content /%**	82	90	90	18	100	20.0
	80	90	90	16	100	17.8
	78	90	90	11	100	12.2
**Volume proportion**	6:3:1	90	90	8	100	8.9
	6:2:2	90	90	18	100	20.0
	7:2:1	90	90	19	100	21.1
**Bulk density (g/ml)**	0.183	90	90	9	100	10.0
	0.223	90	90	5	100	5.6
	0.262	90	90	31	100	34.4
**Total**		270	270	51	100	17

**Fig 7:**
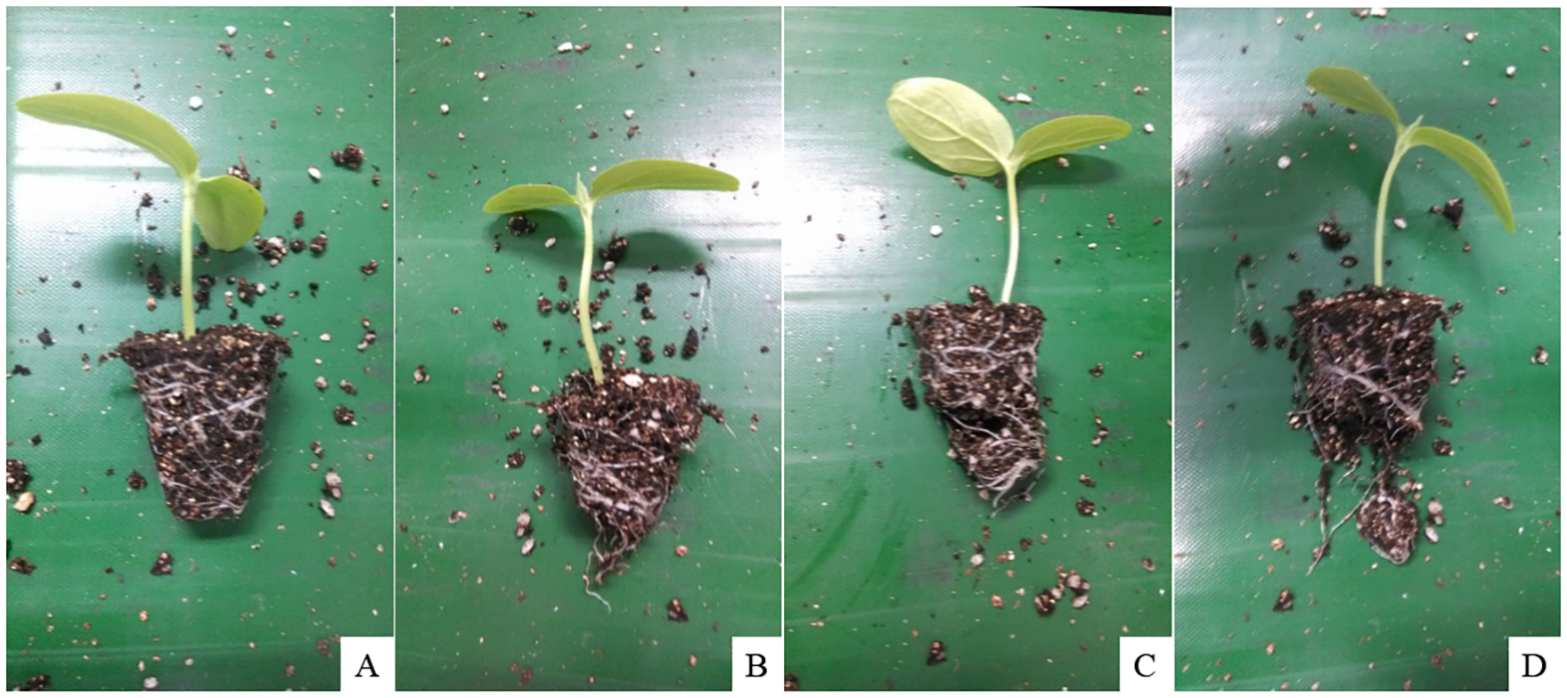
Plug seedlings after transplantation.

Results from Table 4 indicated that all the 270 plug seedlings were successfully transplanted and most plug seedlings were kept intact after transplantation (as shown in Fig 7A). The total transplantation success rate of the end-effector was 100%, and the root plug harm rate was below 17%. There were 50 plug seedlings harmed, most harmed root plugs lost less than 1/5 of substrate or cracked slightly (as shown in Fig 7B or 7C), and only 11 root plugs lost more than 1/5 of substrate (as shown in Fig 7D). The number of harmed seedlings decreased with the decrease of moisture content. Too much water would loosen the root plug. Compared with the other two volume proportions, plug seedlings in trays filled with a peat, including vermiculite: perlite mixture (6:3:1, v/v/v), were less harmed after transplantation. A moderate increase of vermiculite is beneficial for the growth of root systems, which ensure high cohesion of the root plug. High bulk density does not make any better. The results indicated that a medium bulk density (8.5 g/38.2 ml) is good to form firm and cohesive root plugs for automatic transplantation. The harm rate is as low as 7%. Table 5 shows the detailed statistics of harmed root plugs in the experiment. All plug seedlings in Tray 2 and Tray 7 were intact after transplantation. All plug seedlings in the low moisture content region (78%) of Tray 5 and Tray 8 were also intact after transplantation. The results in Tables 4 and 5 indicate that the designed end-effector is very efficient.

**Table 5:** Detailed statistics of harmed root plugs in the experiment.

Tray number	Region 1 (78% moisture content)			Region 2 (80% moisture content)			Region 3 (82% moisture content)		
	Substrate lose (<1/5)	Substrate lose (≥1/5)	Root plug crack	Substrate lose (<1/5)	Substrate lose (≥1/5)	Root plug crack	Substrate lose (<1/5)	Substrate lose (≥1/5)	Root plug crack
**1**	0	0	1	1	0	0	0	0	1
**2**	0	0	0	0	0	0	0	0	0
**3**	0	0	1	1	2	0	0	0	1
**4**	0	1	1	0	0	2	0	1	1
**5**	0	0	0	0	1	0	2	0	0
**6**	0	1	1	1	0	1	0	2	3
**7**	0	0	0	0	0	0	0	0	0
**8**	0	0	0	1	0	0	0	0	1
**9**	0	1	4	0	1	5	0	1	5
**Total**	0	3	8	4	4	8	2	4	12

### Analysis of the extrusion and adhesive forces

**Fig 8:**
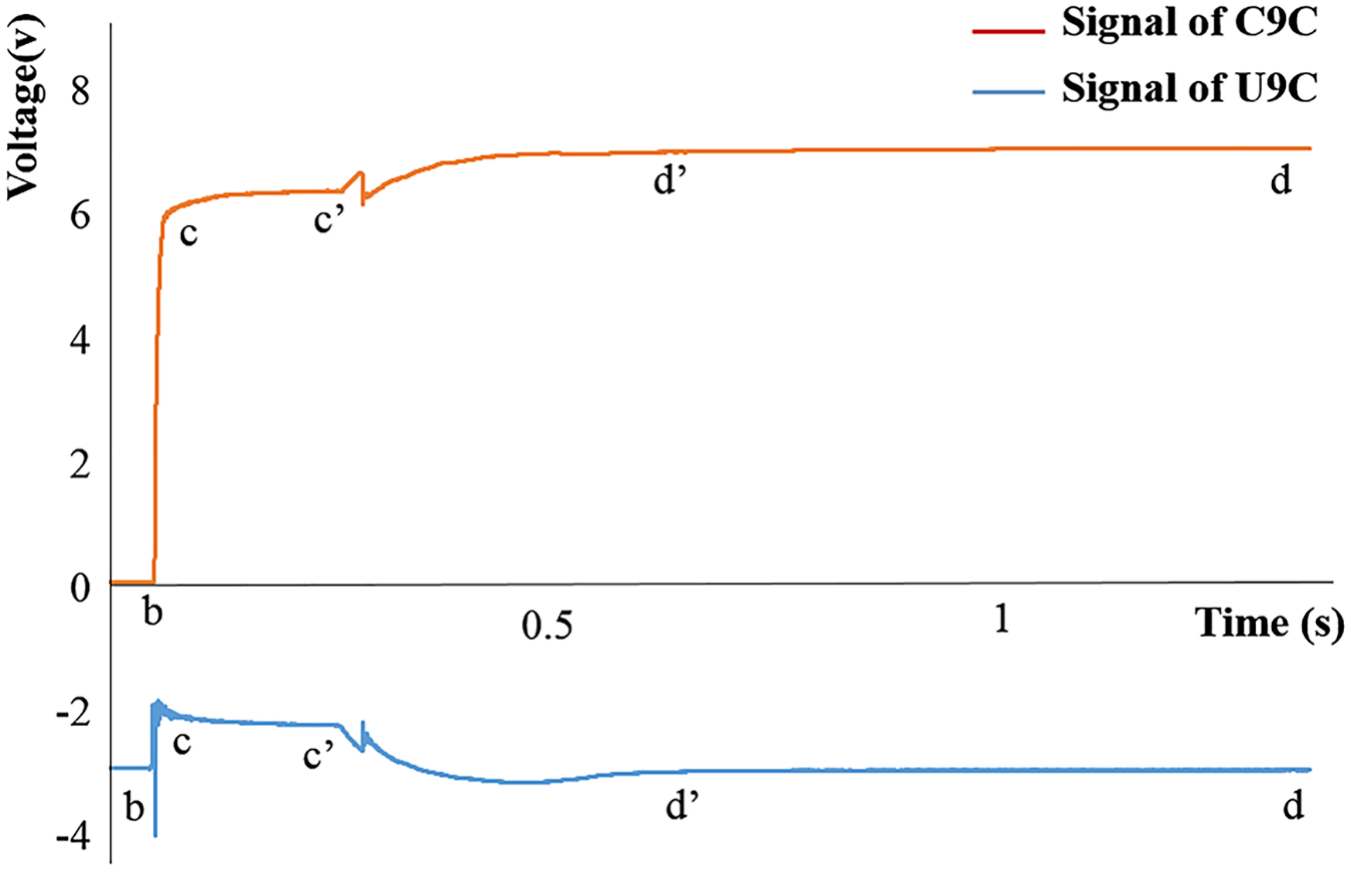
Signals from the C9C and U9C transducers.

Fig 8 shows the typical transplanting process signals of the C9C and U9C transducers. The signals correspond to Step B to Step D of the transplanting process in Fig 2. The signal collection started when the end-effector met the surface of root plugs. The end-effector clamped root plugs (the signal curves from Point b to Point c) and maintenance of the root plugs (the signal curves from Point c to Point c’). The plug seedlings were lifted and separated from the cell (the signal curves from Point c’ to Point d’). After separating, the end-effector continued rising to the height as set before (the signal curves from Point d’ to Point d). According to the linear fitting Eqs (7) and (8), the tension on the U9C transducer (*F*_*U*_) and the stress on the C9C transducer (*F*_*V*_) could be easily obtained. The minimum tension value of the U9C transducer from Point c’ to Point d’ ($F_{Uc\prime d\prime }^{min}$) was used as *F*_*1*_. The stable tension value of the U9C transducer from Point d’ to Point d (*F*_*Vd*′*d*_) was used as the total gravity of end-effector and plug seedling (*G*_*e*_ + *G*_*s*_). Before the experiment, the end-effector ran five times without plug seedlings. The average stress value of the C9C transducer from Point c to Point c’ ($F_{Vcc\prime }^{\prime }$) was used as the total resistance *F*_*s*_. When the end-effector was working with plug seedlings, the average stress value of the C9C transducer from Point c to Point c’ (*F*_*Vcc*′_) was *F*_*2*_. For the experiment, sin *α* = 2/18, *L*_*1*_ = 136 mm, and *L*_*2*_ = 18 mm. The adhesive force *F*_*L*_ and the extrusion force *F*_*k*_ are calculated from Eqs (5) and (6).

Table 6 displays the extrusion force *F*_*k*_, the adhesive force *F*_*L*_ and the value of *F*_*K*_/*F*_*L*_ in the combinational effect trial. Each result was the average value of 10 plug seedlings. Considering the results in Table 5, in each region, if there had any root plug that lost more than 1/5 of substrate or more than one root plug that lost less than 1/5 of substrate or cracked slightly, the transplanting results of this region would be considered poor. The transplanting results would not be considered good when the value of *F*_*K*_/*F*_*L*_ is smaller than 5.99 or larger than 8.67. For a plug seedling at one condition, the adhesive force *F*_*L*_ is also a certain value, but the extrusion force *F*_*k*_ could be changed by adjusting the needles of the end-effector. The root plug would be broken if the extrusion force is too large. On the other hand, too small of an extrusion force could not hold the plug firmly. To guarantee a better transplantation performance, the end-effector was adjusted to make the extrusion force *F*_*k*_ in a reasonable range (5.99–8.67 times the adhesive force *F*_*L*_).

**Table 6:** Adhesive force *F*_*L*_ and extrusion force *F*_*k*_ during the combinational effect trial.

Tray number	Region 1			Region 2			Region 3		
	*F*_*L*_/ N	*F*_*K*_/ N	*F*_*K*_/*F*_*L*_	*F*_*L*_/ N	*F*_*K*_ /N	*F*_*K*_/*F*_*L*_	*F*_*L*_/ N	*F*_*K*_ /N	*F*_*K*_/*F*_*L*_
1	2.14	18.56	8.67	1.78	12.50	7.02	1.57	9.45	6.02
2	2.68	18.46	6.89	2.35	16.02	6.82	2.13	14.75	6.92
3	2.71	18.14	6.69	2.69	15.97	5.94	2.31	14.11	6.11
4	1.82	18.67	10.26	1.60	15.40	9.63	1.41	12.32	8.74
5	2.97	17.78	5.99	2.72	15.56	5.72	2.50	13.07	5.23
6	3.09	16.54	5.35	2.92	14.90	5.10	2.58	13.36	5.18
7	2.02	16.76	8.30	1.86	15.82	8.51	1.60	13.14	8.21
8	2.71	16.55	6.11	2.33	16.26	6.98	2.31	15.03	6.51
9	2.72	15.58	5.73	2.65	15.82	5.97	2.67	15.93	5.97
Average	2.55	17.45	6.84	2.30	15.36	6.68	2.08	13.36	6.42

### Significance test of the three factors based on ANOVA

#### Significance test on the extrusion force

ANOVA is a useful method when the objective is an assessment of the impact of certain controllable factors on a specific response. Analysis of variance for extrusion forces based on ANOVA are presented in Table 7. The results showed that all the three variable factors and their interactions had significant effects on the extrusion force (P < 0.05). Table 8 presents the independent impact of the three variable factors. There were no significant differences between 6:3:1 and 6:2:2 volume proportion of substrate ingredients with respect to extrusion force. The extrusion forces increased when the moisture content of growth substrate decreased. Root plugs would be soft and easily deformed if they contained too much water. Thus, the extrusion force decreased. The extrusion forces increased with the bulk density first and then decreased. Table 5 indicates that root plugs were easily cracked when the bulk density was too large. When the root plugs cracked, the extrusion forces decreased. The mean extrusion force under 7:2:1 volume proportion of substrate ingredients was greater than the other two, which indicated that the increase of peat content enhances the extrusion force.

**Table 7:** Analysis of variance for extrusion forces.

Source	Sum of Squares	df	Mean Square	F	P
**Corrected Model**	1284.408^a^	26	49.400	42.604	<0.001
**Intercept**	63808.250	1	63808.250	55029.865	<0.001
**Moisture content**	751.931	2	375.965	324.242	<0.001
**Volume proportion**	11.947	2	5.974	5.152	0.006
**Bulk density**	86.565	2	43.282	37.328	<0.001
**Moisture content** * **Volume proportion**	164.639	4	41.160	35.497	<0.001
**Moisture content** * **Bulk density**	164.181	4	41.045	35.398	<0.001
**Volume proportion** * **Bulk density**	79.204	4	19.801	17.077	<0.001
**Moisture content** * **Volume proportion** * **Bulk density**	25.941	8	3.243	2.797	0.006
**Error**	281.763	243	1.160		
**Total**	65374.421	270			
**Corrected Total**	1566.171	269			

^a^. R Squared = 0.820 (Adjusted R Squared = 0.801)

* Means interactions of factors before and after it.

**Table 8:** Independent impacts of the three variable factors on extrusion forces (/N).

Factors	Extrusion forces (mean values ± standard deviations)		
**Moisture content /%**	82	80	78
	13.35±2.31^a^	15.40±1.49^b^	17.43±1.27^ab^
**Volume proportion**	6:3:1	6:2:2	7:2:1
	15.32±3.03^a^	15.15±2.58^a^	15.65±1.27^b^
**Bulk density /g/ml**	0.183	0.223	0.262
	14.59±3.42^a^	15.92±1.61^b^	15.60±1.54^ab^

^a,b^ Means in a row with no common superscripts are significantly different (P < 0.05).

#### Significance test on the adhesive force

Analysis of variance for adhesive forces based on ANOVA are presented in Table 9. Results show that all the three factors had significant effects on adhesive force individually (P < 0.05). There was a significant interaction between volume proportion and bulk density (P < 0.05). Table 10 presents the independent impact of the three variable factors on adhesive force. There were no significant differences between 6:3:1 and 7:2:1 volume proportion of substrate ingredients with respect to adhesive force. The adhesive forces increased when the moisture content of growth substrate decreased. A reduction of moisture would increase the adhesion between root plug and tray cell. The adhesive forces increased with the increase of bulk density. The expansion of root plugs would be stronger when the bulk density increased. Thus, the friction between root plug and tray cell increased. The mean adhesive force under 6:2:2 volume proportion of substrate ingredients were greater than the other two. This indicated that the increase of peat content could increase the adhesive force.

**Table 9:** Analysis of variance for adhesive forces.

Source	Sum of Squares	df	Mean Square	F	P
**Corrected Model**	59.448^a^	26	2.286	49.677	<0.001
**Intercept**	1435.024	1	1435.024	31177.986	<0.001
**Moisture content**	10.704	2	5.352	116.280	<0.001
**Volume proportion**	0.957	2	0.478	10.393	<0.001
**Bulk density**	43.175	2	21.588	469.020	<0.001
**Moisture content** * **Volume proportion**	0.197	4	0.049	1.069	0.372
**Moisture content** * **Bulk density**	0.190	4	0.047	1.029	0.393
**Volume proportion** * **Bulk density**	4.059	4	1.015	22.044	<0.001
**Moisture content** * **Volume proportion** * **Bulk density**	0.168	8	0.021	0.456	0.886
**Error**	11.185	243	0.046		
**Total**	1505.657	270			
**Corrected Total**	70.633	269			

^a^. R Squared = 0.842 (Adjusted R Squared = 0.825)

* Means interactions of factors before and after it.

**Table 10:** Independent impacts of the three variable factors on adhesive forces (/N).

Factors	Adhesive forces (mean values ± standard deviations)		
**Moisture content /%**	82	80	78
	2.06±0.47^a^	2.30±0.47^b^	2.55±0.48^ab^
**Volume proportion**	6:3:1	6:2:2	7:2:1
	2.25±0.42^a^	2.39±0.64^b^	2.28±0.44^a^
**Bulk density /g/ml**	0.183	0.223	0.262
	1.75±0.31^a^	2.51±0.31^b^	2.66±0.34^ab^

^a,b^ Means in a row with no common superscripts are significantly different (P < 0.05).

## Conclusion

The newly designed end-effector with four needles was proven to be an efficient end-effector. This end-effector clamped the plug at the same time with the four needles penetrating into substrate and without increasing time consumption. The total transplantation success rate of the end-effector was 100%, and the total harm rate to root plug was 17%. Most harmed root plugs lost less than 1/5 of substrate or cracked slightly. The designed end-effector performed well in all of the combinations of factors. Making the bulk density moderate before nursery and keeping the growth media at a low moisture content before transplantation is better for keeping the root plug intact. The combinational trial showed that all the three variable factors and their interactions had significant effects on the extrusion force. All the three factors had significant effects on adhesive force. The relationship between these two forces and the transplantation performance was established. To guarantee better transplantation results, the end-effector should be adjusted to make the extrusion force *F*_*k*_ in a reasonable range (5.99–8.67 times of the adhesive force *F*_*L*_) before transplantation. This could provide a scientific basis for end-effector application during transplantation.

## Supporting information

S1 File All extrusion force and adhesive force. (XLSX)
